# Epigenetics in liver disease: from biology to therapeutics

**DOI:** 10.1136/gutjnl-2015-311292

**Published:** 2016-09-13

**Authors:** Timothy Hardy, Derek A Mann

**Affiliations:** 1 Fibrosis Laboratories, Institute of Cellular Medicine, Newcastle University, Newcastle upon Tyne, UK; 2 Department of Gastroenterology and Hepatology, The Newcastle upon Tyne Hospitals NHS Foundation Trust, Newcastle upon Tyne, UK

**Keywords:** FIBROSIS, GENE REGULATION, HEPATOCELLULAR CARCINOMA, CHRONIC LIVER DISEASE, CELL BIOLOGY

## Abstract

Knowledge of the fundamental epigenetic mechanisms governing gene expression and cellular phenotype are sufficiently advanced that novel insights into the epigenetic control of chronic liver disease are now emerging. Hepatologists are in the process of shedding light on the roles played by DNA methylation, histone/chromatin modifications and non-coding RNAs in specific liver pathologies. Alongside these discoveries are advances in the technologies for the detection and quantification of epigenetic biomarkers, either directly from patient tissue or from body fluids. The premise for this review is to survey the recent advances in the field of liver epigenetics and to explore their potential for translation by industry and clinical hepatologists for the design of novel therapeutics and diagnostic/prognostic biomarkers. In particular, we present findings in the context of hepatocellular carcinoma, fibrosis and non-alcoholic fatty liver disease, where there is urgent unmet need for new clinical interventions and biomarkers.

## Introduction

Irrespective of aetiology, the progression of chronic liver damage and inflammation to cirrhosis and/or cancer is highly variable between individuals. If liver damage persists, a large percentage of patients can expect their disease to advance over their lifetime. However, the course and rate of progression of their disease is often unpredictable using prognostic tools currently available in the clinic. As an example, a recent study of paired biopsies from 108 patients with non-alcoholic fatty liver disease (NAFLD) revealed that 42% patients had fibrosis progression, 40% had no change in fibrosis, while 18% had fibrosis regression; 9% underwent progression from bland steatosis to fibrosing-steatohepatitis.^1^ However, the degree of progression was variable, ranging from one to three stages, and the molecular basis for this variability remains poorly defined. While genome-wide association studies (GWAS) have identified genetic variants that show association with fibrosis and/or hepatocellular carcinoma (HCC),^2^ ^3^ to date their predictive value with regard to outcomes in clinical practice have been limited. Furthermore, the biological function of variants remains challenging to elucidate and direct patient benefit appears distant. It is well established that there are numerous non-genetic factors that influence the progression of liver disease, including patient age, sex, body composition, diet, exercise, microbiome, alcohol consumption and their history of smoking.^4^ The study of epigenetics is, in essence, the investigation of how non-genetic factors act upon the genome to influence gene expression and phenotype. Epigenetics can therefore enable us to interrogate the mechanisms that underlie disease phenotype, and it is hoped to shed new light on the basis for interpatient variability in disease progression. Furthermore, the highly dynamic nature of epigenetic mechanisms and their regulators in response to environmental cues offers hope for the advent of epigenetic therapies in liver disease, as is now occurring in clinical oncology. However, this dynamism, coupled with the complexity of epigenetic mechanisms that can operate both locally at the gene level as well as globally across the epigenome, presents significant challenges. Improvements in next generation sequencing technologies and their ability to generate genome-wide quantitative data are helping to meet this challenge. As an example, DNA methylation can now be quantified in a sequence-specific manner across the entire genome to generate a methylome map, and there is potential to carry this out on either single cells or circulating free DNA.^5^ ^6^ Similarly, emerging omics approaches to the study of histone modifications will offer unparralled insights into the functional associations of alterations in the chromatin landscape of cells and disease processes. In this review, we will describe the basic epigenetic players and mechanisms before discussing recent important discoveries in liver epigenetics.

## Epigenetics and the case for its study in liver disease

Conrad Waddington established the field of epigenetics in 1942 when he proposed an uncoupling of genotype and phenotype, implying that regulatory processes linked the two.^7^ The first conclusive evidence that cellular phenotype is dictated by mechanisms other than that encoded within the DNA sequence came from classic experiments in frogs, for which Sir John Gurdon was later awarded the Nobel prize. He demonstrated that transplantation of a nucleus from a fully differentiated somatic cell into a denucleated egg supported the development of a tadpole. The conclusion was that the genome sequence is stable through development and is not per se determining phenotype; rather the latter is dictated by developmentally programmed patterns of gene expression that are responsive to the cellular microenvironment.^8^ The mechanisms that regulate this flow of genetic information include the actions of key transcription factors (as demonstrated by the experiments of Shinya Yamanaka, who shared the 2012 Nobel prize with Gurdon) that operate in concert with epigenetic factors to modulate the rate at which individual genes are transcribed into RNA. Critically, the activities of epigenetic and transcription factors are plastic and highly dynamic, being influenced by the metabolic state of the cell and its ever changing extracellular environment. In turn, even the most differentiated population of nucleated cells will display phenotypic heterogeneity and have the potential for behavioural reprogramming. The normal healthy functions of the liver, as with any tissue, are critically dependent upon robust maintenance of the behavioural properties of its constituent cells. Unhealthy perturbation of liver function reflects a loss of homeostasis, whereby the constituent liver cells fail to maintain their distinct phenotypic characteristics in the face of challenges in the local microenvironment induced by metabolic, xenobiotic, immune or microbial triggers. Hence, by illuminating the epigenetic modifications that are associated with specific pathologies such as cirrhosis or cancer we can better understand the molecular basis for loss of cellular homeostasis in chronic liver disease (box 1).

Box 1 **An epigenetic glossary**

5-hydroxymethylcytosine: the oxidation of 5-methylcytosine modified DNA, by the Tet family of enzymes.

5-methylcytosine: an epigenetic modification of DNA occurring at CpG dinucleotides.

Bisulfite conversion: the selective deamination of unmethylated cytosine bases to uracil by treatment with sodium bisulfite; used for methylation analysis.

Chromatin: the formed complex of DNA and histones required for nuclear compaction.

CpG islands: regions of DNA enriched for CpG dinucleotides; CpG islands are >200 bp long, located at transcriptional start sites, and predominantly unmethylated.

Differentially methylated region: regions of DNA in an organisms genome that is methylated differentially between disease phenotypes.

DNA methylation: an epigenetic modification in which a methyl group is covalently bonded to the 5th carbon of the cytosine pyrimidine ring in a CpG dinucleotide, frequently (but not exclusively) associated with gene silencing.

DNA methyltransferases: a group of enzymes that catalyse the addition of a methyl group to DNA. Members include DNMT1 required for maintenance of DNA methylation and DNMT 3a/b involved in de novo methylation.

Euchromatin: unpacked chromatin, allowing access for transcription factors and gene expression.

Heterochromatin: compacted chromatin, inaccessible to transcription factors, containing poorly expressed genes.

Histone acetyltransferase: enzyme that acetylates histones at specific lysine residues.

Histone deacetylase: enzyme that remove acetyl groups from *N*(6)-acetyl-lysine residues on a histone.

Histone: the core protein around which DNA is wound tight, ordering DNA into nucleosomes. Core histones include H2A, H2B, H3 and H4.

Histone modification: post-translational modifications of histones including the addition or removal of methylation, acetylation, phosphorylation, ubiquitination, sumoylation and marks.

Histone variants: variant proteins that can be inserted into nucleosomes, and may have intrinsic gene regulatory functions.

Long non-coding RNA: non-protein-coding RNA>200 nucleotides with gene regulatory properties. Examples include Xist, HOX transcript antisense intergenic RNA, highly upregulated in liver cancer and high expression in hepatocellular carcinoma.

microRNA: Small non-protein coding RNA (∼22 nucleotides) that regulate cellular processes by controlling transcription and translation of mRNA.

Non-coding RNA (ncRNA): the majority of the genome is transcribed into non-protein encoding RNA, involved in many cellular processes. Examples include microRNAs, small interfering RNAs (siRNAs), Piwi-interacting RNAs, long non-coding RNAs (lncRNAs) and long intergenic RNAs.

Nucleosome: the basic structural unit of chromatin, allowing compaction; one nucleosome is comprised of 147 bp of DNA wrapped around a histone octamer including two molecules each of the core histones H2A/B, H3 and H4.

## The major epigenetic mechanisms

### DNA methylation

DNA can be covalently modified and the best-known modification is methylation of cytosine at its 5th carbon ring, which is mainly, although not exclusively, found at cytosines within CpG dinucleotides. The most well-established role of the me-CpG mark is when it occurs at high density within the so-called CpG islands which predominantly traverse 5′ promoter regions; methylation at these regions results in strong transcriptional repression. Importantly, methyl-CpG is a signal for recognition of DNA by specific proteins containing a so-called methyl binding domain (MBD). The MBD family (MBD1, MBD2, MeCP2 and MBD4) mediate transcriptional repression at CpG islands.^9^ The majority of methylated CpG islands are developmentally established and become stable in the adult. However in cancer, methylation can be acquired at normally unmethylated CpG islands and is usually accompanied by a second repressive epigenetic mark, methylation of histone H3 at its lysine 27 residue, which compacts chromatin and inhibits transcription.^10^ Genome-wide, single-base resolution mapping of DNA methylation has revealed that there is considerable dynamic turnover outside of CpG islands and suggests that the occupancy of transcription factors at these CpG sites is associated with loss of methylation.^11^ ^12^ Despite this knowledge, there is at present inconclusive evidence that loss of methylation outside CpG islands instructs transcriptional information at a local level. Further insights may emerge now that the enzymes that instruct DNA methylation (DNA methyltransferases: DNMT1, DNMT3a and DNMT3b) and demethylation (ten-eleven translocation enzymes [TET1–3]) are beginning to be functionally defined. DNMT1 is a maintenance methylase that during cell division recognises a hemimethylated site on a new DNA strand and regenerates the bimethylated state.^13^ In this way, CpG methylation patterns are faithfully maintained in daughter cells. However, de novo methylation does occur in the absence of cell division and is regulated by DNMT3a and DNMT3b.^14^ The TET enzymes catalyse the step-wise oxidation of methyl groups on DNA leading to the eventual restoration of the unmodified cytosine residue.^15^ Experimental deletion of the TET enzymes results in increased methylation at gene enhancers and subtle alterations in the expression of genes linked with these enhancer regions.^16^ ^17^ Turnover of DNA methylation may therefore be an ongoing process in most cell types and has potential to be modulated via changes in the relative expression of DNMT and TET proteins. The discovery that vitamin C can enhance TET activity in cells supports this idea and indicates the existence of mechanisms for modulating DNA methylation in response to environmental cues.^18^

### Histone modifications and chromatin remodelling

In order to achieve the feat of compaction required for 2 m of DNA to be condensed into a human nucleus, DNA interacts with specialised proteins to form tightly packed chromatin; for chromosomal processes to occur such as gene transcription, this must be iteratively unpacked and repacked in a regulated manner, providing an opportunity for dynamic gene regulation. The most basic level of chromatin packing is known as the nucleosome, each core particle consisting of 147 bp of double stranded DNA wrapped around a complex of eight histone proteins (two copies each of H2A, H2B, H3 and H4) seen under an electron microscope as ‘beads on a string’; linker DNA being the *string* and the *beads* representing the nucleosome core particle. Each of the core histones has an unstructured N-terminal amino acid tail extension that can be subject to a plethora of covalent, post-translational modifications that control aspects of chromatin structure and function, either directly affecting chromatin structure or comprising signals to be recognised by protein effectors.^19^ Histones can be acetylated, methylated on lysine and arginine, phosphorylated on serine, ubiquitinated, sumoylated and ADP-ribosylated. Histone acetylation loosens chromatin to transcriptionally active *euchromatin*. By contrast, trimethylation of lysine 9 of histone 3 (H3K9me3) and H3K27me3 are associated with condensed, transcriptionally silent *heterochromatin.* However, histone lysine methylation can also promote transcription depending on which lysine is modified for example, H3K4me3 and H3K36me2/3 are generally associated with euchromatin. Variants of the core histones (except H4) can be inserted by ATP-dependent chromatin-remodelling complexes and regulate nucleosome structure. As an example, exchange of H2 for H2A.Z is important for gene expression,^20^ while exchange for macroH2A is associated with transcriptional repression.^21^ Histone modifications are highly dynamic, and regulated by ‘writer’ and ‘eraser’ enzymes that add or remove post-translational modifications, respectively. They can serve as marks for recruitment of ATP-dependent chromatin remodelling complexes such as switch/sucrose non-fermentable (SWI/SNF) that remodel nucleosome and chromatin structure allowing access to gene regulatory proteins; mammalian SWI/SNF can slide nucleosomes on DNA or can exchange or extrude histones, promoting gene activation.^10^ In contrast, repressive chromatin remodellers act on nucleosomes to form densely packed chromatin, restricting access to transcription factors and recruiting other chromatin modifiers that help impose repression. Polycomb group proteins are found in two multiprotein complexes known as polycomb repressor complexes 1 and 2 (PRC1 and PRC2) and play a major role in cell differentiation. PRC2 regulates chromatin structure, in part by H3K27 trimethylation through its enzymatic subunit EZH2. The PRC1 complex monoubiquitylates H2AK119 via the ubiquitin ligases RING1A and RING1B; PRC1 can also bind to H3K27me3 formed by PRC2 catalysis and both act to repress gene expression.^22^

### Non-coding RNAs

A large amount of the transcribed genome is structured such that it is not destined to be translated into proteins but instead carries out regulatory functions in RNA forms known as non-coding RNAs (ncRNAs).^23^ MicroRNAs (miRNAs), which are sequence-specific 22-nucleotide RNA molecules, are by far the most intensively studied and functionally best characterised ncRNAs. The mechanism by which miRNA regulate gene expression is to modulate the translation of their target mRNAs and this usually results in downregulation of protein expression. A number of key regulatory miRNA have already been developed as targets for antiviral^24^ and anticancer drug targets^25^ and are implicated in the control of liver fibrosis.^26^ Many other classes of ncRNA have been discovered including ribosomal RNAs, ribozymes, endogenous small interfering RNAs, Piwi-associated RNAs and long non-coding RNAs (lncRNAs). Readers are referred to an excellent recent review on these molecules.^27^ LncRNAs are >200 nucleotides in length that can be further classified into antisense lncRNAs that overlap known protein-coding regions, intronic lncRNAs, overlapping transcripts and long intergenic RNAs encoded in the intergenic space between protein-coding regions. LncRNAs are implicated as regulators of a wide variety of biological processes relevant to liver homeostasis and disease including cell proliferation, differentiation, migration and survival, yet surprisingly for the majority of lncRNAs their precise mechanisms of action are obscure. The best-studied lncRNA function is in X chromosome inactivation in which the 17 kb transcript *Xist* recruits repressive epigenetic factors such as PRC2 to bring about effective repression of gene transcription and ensure appropriate X chromosome gene dosaging in females.^28^ The lncRNA *Tsix* can recruit Dnmt3a, while *Kcnq1ot1* recruits Dnmt1; these observations indicating that lncRNAs may act as guides for bringing CpG methylases to specific sites in the genome.^29^ ^30^ lncRNAs may therefore reveal how DNA methylation and histone modifications are annotated in a gene-specific and sequence-specific manner during and after development. However, the actions of lncRNAs extend to many other biological processes including the regulation of transcription factor binding, mRNA processing, mRNA stability, protein translation and signal transduction.^23^

## Epigenetic mechanisms in HCC

### DNA methylation and HCC

Typical epigenetic lesions in human cancer include a genome-scale loss of DNA methylation, with loci-specific de novo hypermethylation at gene promoters of tumour suppressor genes (TSGs) leading to transcriptional repression of downstream TSGs; such aberrations may have potential as diagnostic markers in the progression of HCC. There are many studies reporting HCC-specific DNA methylation changes, notably some at a genome-wide level.^31–35^ Of these, the largest study to date profiled the DNA methylation landscape in 221 patients with HCC treated with surgical resection of a predominantly viral aetiology (66%). In this study, 485 000 CpG dinucleotides were interrogated using methylation array technology; a 36-probe hypermethylation signature, termed a DNA methylation mortality index (MI), accurately predicted survival in patients with HCC, independent of known clinical and pathological risk factors for survival (HR: 13.35; 95% CI 7.94 to 22.42; p<0.001).^36^ DNA methylation MI was also an independent predictor of overall recurrence (HR: 5.8; 95% CI 3.1 to 11; p<0.001). This was validated in an independent cohort of predominantly alcohol-related, aggressive tumours. Combined with transcriptomics, the study identified mRNA signatures enriched with progenitor cell features such as epithelial cell adhesion molecule (EpCAM) and S2 (p=0.009 and p=0.006, respectively) in the DNA methylation MI high group.^36^ The landscape of aberrant methylation in human HCC displayed general hypomethylation compared with normal liver, mainly located in the intergenic (39.9%) and body regions (34.5%), whereas hypermethylated probes were generally in promoter areas (50.5%). In relation to CpG islands, hypermethylation occurred at the islands and shores (63.9% and 24.8%), whereas hypomethylation were mainly in open sea regions (63.55%).^36^ Gene ontology analysis showed the top-ranked probes included genes related to expression regulation, and deregulated signalling cascades such as insulin growth factor (IGF), phosphatidylinositol 3-kinase, transforming growth factor β (TGF-B), WNT signalling and cadherin.^36^ Finally, the study demonstrated aberrant methylation in known and candidate epidrivers of disease. Hypermethylation was confirmed, as demonstrated in prior studies, in well-known TSGs such as *RASSF1*, *APC*, *NEFH*^34^ and *NOTCH3;*^37^ candidate epidrivers included *SEPT9* a tumour suppressor in colon and ovarian cancer.^38^ This recent study, when added to previous genome-wide DNA methylation studies, confirms the crucial role of deregulated DNA methylation in human HCC development and critically survival outcome.

Despite much enthusiasm for genome scale epigenetic studies in HCC, there remains a clear caveat when interpreting the clinical and biological meaning of such data. Critically, cancerous tissue represents a heterogeneous mix of tumour cells, fibroblasts, stromal cells, inflammatory cells and non-parenchymal cells. Thus, any methylation signatures may simply be due to changes in numbers of cells and constituents, rather than direct methylation changes in cancer cells, relevant to cancer biology. Further genome-wide studies should utse technologies such as laser capture microdissection or high-speed cell sorting to separate tumour cells from contaminating cells.

The functional relevance of aberrant DNA methylation needs to be rigorously assessed. Only a handful of studies have tested potential aberrantly methylated TSGs in vivo*;*^34^ ^39^ ^40^ in a recent study, 71 HCC samples were subjected to low density methylation array, after exposure to 5-aza-2′-deoxycytidine to reverse DNA methylation. Of 13 candidate TSGs, 2 (*SMPD3* and *NEFH*) were further functionally tested; knocking down these genes with small hairpin RNA promoted cell invasion and migration in vitro, and increased tumour formation after subcutaneous injection or transplantation into mice.^34^ Reduced levels of *SMPD3* were associated with early recurrence after curative resection.^34^ Although these data provide good evidence for a functional role of DNA methylation and gene expression in candidate TSGs, ideally, direct functional correlation between aberrant methylation and altered gene expression requires in vivo loci-specific manipulation of DNA methylation and demonstration of a correlation with a change in gene expression; such studies are greatly anticipated.

Preliminary studies are beginning to show the feasibility of measuring circulating cell-free tumour DNA (cfDNA) methylation for early diagnosis of HCC. In one such study, plasma DNA had detectable hypermethylation (>5%) for *CDKL2, CDKN2A, HIST13G, STEAP4* and *ZNF154* in 37%–63% of patients with HCC (n=38).^33^ Clearly, further studies with larger patient cohorts are needed to fully assess the utility of this approach, although similar results have been found in other solid organ cancers.^41^ Finally, tumour methylation patterns can be detected in cfDNA and short cfDNA fragments can harbour footprints of transcription factor binding. This information correlates with tissue nucleosome occupancy and transcription factor binding, informing the cell type of origin of cancers such as the liver,^42^ providing another non-invasive epigenetic approach to disease monitoring.

### Chromatin remodellers and HCC

Perhaps the best-studied deregluated master chromatin remodeller in HCC is EZH2, the catalytic subunit of the PRC2 complex. EZH2 is overexpressed in HCC and is associated with malignant progression, vascular invasion and cell proliferation.^43^ A progressive increase in EZH2 protein expression from dysplastic nodule to early HCC is associated with advanced HCC.^44^ EZH2 protein expression can serve as a biomarker distinguishing preneoplastic lesions from HCC (area under the receiver operating characteristic (AUROC)=0.935) and positive expression of EZH2 correlates with reduced survival.^45^ In vivo studies in nude mice show a key role for EZH2 in HCC tumourigenesis; intratumoral EZH2 knockdown produced significant tumour regression and suggest EZH2 as a promising target for therapeutics.^46^ EZH2 was found to repress several negative regulators of the Wnt pathway, activating β-catenin-mediated cellular proliferation.^47^ A recent study found that EZH2 and its structural partners EED, SUZ12 and RBP7 contribute to tumourigenesis by silencing multiple tumour suppressor miRNAs^48^ including miR-101, a recently characterised negative regulator of both EZH2 and EED.^49^ EZH2 also regulates expression of the chemokine receptor CXCR4 by controlling expression of its regulator miR-622 and patients with overactivity of this pathway have a worse overall survival.^50^

Mutational exome screening of viral-related and alcohol-related tumours revealed recurrent inactivating alterations in *ARID1A* and *ARID2* in 15% and 5% of tumours, respectively;^51^ ^52^ both proteins are part of the SWI/SNF ATPase-related chromatin-remodelling complex, shown to have tumour suppressor functions.^53^ Exome sequencing in 87 HCCs and matched non-tumour tissue of viral origin revealed missense gene mutations encoding H3K4 methyltransferases *MLL1, MLL2, MLL3* and *MLL4.* The functional effect of these mutations on histone methylation are yet to be validated, but these enzymes are important transcriptional coactivators required for expression of p53 target genes subsequent to DNA damage.^54^

### ncRNAs in HCC

**Figure 1:**
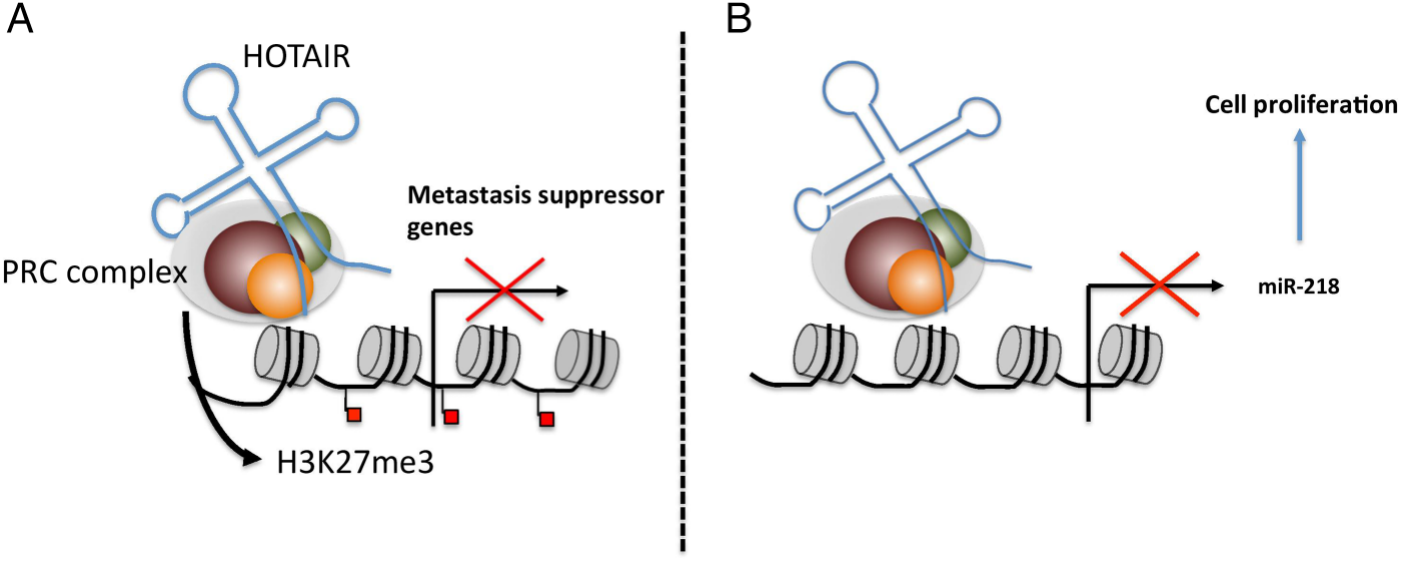
HOTAIR-mediated carcinogenesis. Hotair acts a guide, (A) redirecting the PRC (EZH2, SUZ12, EED) and H3K27me3 patterns favouring tumour metastasis and (B) silencing of miR-218 that promotes cell proliferation through aberrant activation of Bmi-1. HOTAIR, HOX transcript antisense intergenic RNA; PRC, polycomb repressor complex.

Of the ncRNAs it is unsurprising that miRNAs are the most extensively studied in HCC, for this class of ncRNAs readers are referred to a recent detailed review.^55^ By contrast, only a handful of lncRNAs have been validated and functionally characterised as important in regulating the biology of HCC. Highly upregulated in liver cancer (HULC), a 500 nt transcript discovered by cDNA microarray is upregulated 33-fold in HCC.^56^ siRNA knockdown of HULC deregulates a number of proliferation-related and HCC-related genes. Of note, HULC RNA is detected in peripheral blood cells of patients with HCC, raising the possibility of novel biomarker.^56^ Further functional clarification in hepatoma cells showed that HULC acts by promoting lipogenesis through ACSL1 activation, disturbing the Clock circadian regulator/brain and muscle arnt-like protein-1 complex^57^ ^58^ and promoting angiogenesis.^59^ HOX transcript antisense intergenic RNA (HOTAIR) is a 2158 nt PRC2-interacting lncRNA upregulated in HCC that is predictive of tumour recurrence in liver transplant patients.^60^ Knockdown of HOTAIR in hepatoma cell lines sensitised to TNFα-induced apoptosis and reduced cell viability.^60^ A recent study validated the upregulation of HOTAIR in HCC specimens and siRNA knockdown of HOTAIR inhibited cell growth, induced cell cycle arrest and suppressed tumourigenesis.^61^ Mechanistically, HOTAIR negatively regulates miR-218 by recruiting EZH2 to its promoter; this results in overexpression of Bmi-1, a target of miR-218 and subsequent inactivation of p14^ARF^ and p16^Ink4a^, two TSGs^61^ (see figure 1).

High expression in hepatocellular carcinoma (HEIH) was discovered after genome-wide screening HBV-related HCCs by microarray and is known to interact with EZH2.^62^ High expression of this lncRNA was associated with worse patient survival, and knockdown of HEIH resulted in upregulation of PRC2 target genes such as *p16^Inka.^*^62^ Next generation sequencing studies are expanding the list of lncRNAs involved in HCC; in a recent study a number of novel lncRNAs were found to be differentially expressed in HCC.^63^ However, known deregulated lncRNAs in HCC, HULC and H19, were not validated in this study.^63^ Future studies using next generation sequencing will certainly illuminate the role of lncRNAs in hepatocarcinogenesis and are greatly anticipated.

#### Direct oncogenic roles of viruses by epigenetic deregulation

Viral-aetiology tumours can provide understanding of the relationship between epigenetic dysregulation and cancer biology. Viral proteins, such as HBx, can affect chromatin and transcriptional control promoting regional hypermethylation and global hypomethylation. HBx can conduct targeted deregulation of DNMT1, DNMT3A1 and DNMT3A2, increasing their activity and repressing *IGF3* and *p16^INK^*, through de novo hypermethylation; global hypomethylation of satellite 2 repeat sequences was induced by decreasing DNMT3B.^64^ Another study showed HBx-directed recruitment of DNMT3A to *IL4R* and *MT1F* promoters and subsequent de novo hypermethylation.^65^ A further proposed mechanism for HBx is to enhance DNMT1 expression through downregulation of its repressor miR-152.^66^ Silencing of miR-152 results in global DNA hypermethylation and increases methylation levels at two tumour suppressor genes, *GSTP1* and *CDH1.*^66^ HBx can also stimulate active demethylation and derepression of the EpCAM gene, known to be observed in HBV-aetiology HCC. The NF-κB transcription factor RelA directs this effect by the formation of a DNA demethylation complex comprising TET2, the catalytically inactive DNMT3L and EZH2.^67^ HCV can also directly alter DNA methylation. Gadd45β controls cell cycle, growth arrest and DNA repair and is under expressed in HCV-tumour tissue. The Gadd45β promoter is hypermethylated in HCV transgenic mouse liver and in cells infected with the JFH1 strain of HCV.^68^ Chimeric mice with humanised livers showed time-dependent, genome-wide changes in DNA methylation after intravenous injection with HBV or HCV.^69^ Importantly, a number of common genes were methylated in this mouse model when compared with human HCC samples indicating that the model may be an important tool for interrogating clinically relevant virus-induced epigenome remodelling.^69^

Viruses can also directly alter epigenetic reprogramming linked to oncogenic transformation. In both cellular and animal models, and HBV-related HCC, HBx induces the PLK1-dependent proteosomal degradation of repressive chromatin regulating proteins, SUZ12 (a component of PRC2) and ZNF198 by ubiquitination, leading to overexpression of hepatic stem cell cancer-related genes such as EpCAM; lncRNA HOTAIR seems to act as a ubiquitination scaffold.^70^ HBV integration into the host genome can generate a viral-human hybrid RNA transcript that acts as a lncRNA; HBx-LINE1, a fusion human LINE1 and HBx gene has been identified in HBV-positive HCC and can drive migration and invasion of tumour cell lines by epithelial to mesenchymal transition and nuclear localisation of β-catenin.^71^ A recent study demonstrated that HBxLINE1 promotes carcinoma progression by sequestering miR-122 as a molecular sponge.^72^

## Epigenetics and liver fibrosis

Liver fibrosis is defined by the excessive accumulation of extracellular matrix and scar formation, in the context of chronic damage to the liver. Eventually regenerating hepatocyte nodules form, defining the progression to cirrhosis; hepatocellular cancer and liver failure can ultimately develop with transplantation the only treatment.^73^ However, the progression to end-stage disease is not linear, but nuanced in that only a small minority of patients progress to severe disease. At the heart of this lies an interplay between host genetics or mutations and environmental factors, determining the phenotype of the patient in response to chronic liver injury. Recent studies have provided evidence that DNA methylation is a critical event governing the molecular events underpinning fibrogenesis and disease progression.

Hepatic stellate cell (HSC) transdifferentiation, from a vitamin A storing pericyte, to a profibrogenic myofibroblastic phenotype, is a pivotal event in fibrogenesis; it occurs in response to a variety of external stimuli such as inflammation and hepatocellular damage. Transdifferentiation requires triggering and orchestrating epigenome-wide reprogramming to suppress adipogenic differentiation factors, de novo expression of myofibroblast regulating transcription factors, and cell cycle entry. HSC activation can be inhibited both in vitro and in vivo by small molecule epigenetic inhibitors such as the DNMT1 inhibitor 5-azadeoxycytidine and the EZH2 inhibitor 3-deazaneplanocin A, suggesting a role for DNA methylation and histone modifications in the control of the global alterations in gene expression required for activation.^74^ ^75^ Our laboratory described an epigenetic relay pathway requiring activation in order to drive HSC transdifferentiation. Mice lacking the DNA methylation reader MeCP2 are attenuated in their liver fibrosis and *mecp2*-deficient HSC show reduced levels of fibrogenic markers collagen 1, TIMP-1 and aSMA.^74^ PPARγ expression must be silenced for HSC to activate and acquire their myofibroblast phenotype and this is achieved by two concurrent epigenetic mechanisms. First, methyl-CpGs within the promoter of PPARγ recruit MeCP2, which then directs repressive H3K9me3-modifying enzymes to suppress initiation of transcription. Second, transcriptional elongation is suppressed by EZH2-mediated H3K27me3 modifications in the downstream coding region of PPARγ, with de novo expression of EZH2 being dependent on MeCP2.^74^ However, MECP2 can also stimulate transcription of multiple profibrogenic genes through its control of ASH1, an H3K4/H3K36 histone methyltransferase that activates transcription.^76^ Hence, MeCP2 is a master epigenetic orchestrator of HSC activation.

Increased DNA methylation during HSC activation occurs at the phosphatase and tensin homolog tumour suppressor ad Patched 1, correlating with its reduced expression in HSCs.^77^ This suggested that DNA methylation is subject to turnover during HSC activation. This concept was confirmed and extended in a recent study in which the expression of DNMT and TET enzymes were analysed in both animal models and human liver disease. Experimental fibrosis was associated with decreased expression of TET2 and TET3 and was accompanied by loss of 5-hmC.^78^ In contrast, DNMTs were generally induced at the protein level in fibrotic livers, although this was not accompanied by a change in the global amount of 5-mC.^78^ Furthermore, in mechanistically distinct human fibrotic liver disease, common changes were seen and correlated with global epigenetic changes in experimental liver fibrosis. Both culture-activated and ex vivo purified activated HSCs displayed decreased levels of 5-hmC and increased expression of the de novo DNA methyltransferases DNMT3a/b, consistent with changes seen in fibrotic human and rodent liver.^78^ An important step in this study was genome mapping of sequence-specific alterations in 5-mC and 5-hmC densities during HSC activation by reduced representation bisulfite sequencing. Quiescent and activated HSC displayed distinct 5-mC landscapes across chromosomes 13 to 19, 20, 21 and also of note at the Y chromosome.^78^ The 5-hmC landscape for activated HSC was of lower density across the genome, although chromosome 9 had a particularly high density of 5-hmC modifications that were unique to activated HSC.^78^ Thus, HSC activation is underpinned by genome-wide alterations in DNA methylation that are most likely driven by increased expression of DNMTs and concomitant reduced expression of demethylases.^78^ The next challenge will be to determine if methylome remodelling is a direct regulator of HSC phenotype or is merely a consequence of HSC activation.

As alluded to earlier, histone modifications are induced in HSC transdifferentiation and liver toxins can influence the activities of chromatin remodellers. Hepatocytes cultured with free fatty acids overexpress the ATP-dependent chromatin remodelling proteins Brg1 and Brm, stabilising NF-κB, and this is required for the development of steatosis, inflammation and fibrosis in methionine-choline deficient diet fed mice.^79^ We recently showed transdifferentiating HSCs cultured directly with ethanol show altered expression of multiple epigenetic regulators and display an altered chromatin structure.^80^ MLL1, a H3K4 methyltransferase associated with activation of transcription, was induced by ethanol and recruited to the elastin gene promoter which was associated with enrichment of the H3K4me3 mark at this gene and enhanced expression of elastin.^80^ A prior study showed that acetylation of H3K9, a transcriptionally active modification is induced in a time-dependent and dose-dependent manner in HSCs cultured with ethanol.^81^ These observations highlight the plasticity of the HSC epigenome, its potential to be modulated by environmental factors and if studied further can provide new mechanistic insights into how the course of fibrogenesis may be influenced by xenobiotics and diet.

The progression of chronic human liver disease represents an increase in fibrotic scar deposition and the out-pacing of liver healing by regeneration. Currently, very little is known about the epigenetic control of liver regeneration. However, a recent study showed that loss of *Arid1a*, a component of the chromatin remodelling complexes SWI/SNF, resulted in improved liver regeneration after partial hepatectomy in mice.^82^ Conditional overexpression of *Arid1a* reduced proliferation in wild type (WT) animals after chemical injury. The molecular mechanisms underlying *Arid1a*-mediated impaired regeneration involve chromatin remodelling, ultimately altering transcriptional access by C/EBPα and E2F4, transcription factors that support terminal differentiation and antagonise proliferation.

Many studies have documented the differential expression of numerous miRNAs in HSCs; the biological function of these miRNAs has been less well studied. However, a recent study employed genome-wide technology to identify the miR-29 family as a fibrogenic regulator in carbontetrachloride-injured and bile duct ligated-injured mice.^83^ The expression of the miR-29 family was downregulated in these models of experimental fibrosis, and in human liver tissue and serum from patients with advanced liver disease. Overexpression of miR-29 in HSC reduced collagen expression, moreover TGF-β repressed miR-29 expression.^83^ However, in vivo studies confirming a regulatory function for miR-29 in liver disease are still lacking. connective tissue growth factor (CTGF) is a well-known soluble stimulator of fibrogenesis and is upregulated during HSC activation, along with a reciprocal downregulation of miR-214 which directly binds the CTGF 3′-untranslated region (UTR).^84^ Of note, it was also demonstrated that miR-214 is transported by exosomes and can be delivered to HSCs resulting in the suppression of CTGF 3′-UTR activity, reduced expression of CTGF and inhibition of profibrogenic genes that lie downstream of CTGF.^84^ Circulating miR-214 was reported to be decreased in mice with experimental liver fibrosis, but its presence in circulating exosomes in this model was not assessed.^84^ While numerous miRNAs have been described as regulators of HSC phenotype and fibrosis progression, studies investigating the functions of lncRNAs are awaited.

## Epigenetics and the progression of non-alcoholic steatohepatitis

Non-alcoholic steatohepatitis (NASH) represents the more severe disease entity of NAFLD, the most common liver disease in the Western world due to its association with obesity and type II diabetes. NASH is typically characterised by steatosis, ballooning hepatocytes and lobular inflammation, with or without fibrosis. As with other causes of liver disease, only a minority of patients with NASH progress to advanced fibrosis, cirrhosis and/or HCC. Progression of NASH is governed by host genetics, and the interaction of environmental factors.^4^ Recent studies have shown that the presence of fibrosis on biopsy is the key histological determinant of long-term prognosis.^85^ ^86^ Particular excitement surrounds emerging translational studies that indicate an involvement of DNA methylation in progression to fibrosis. We have shown, in a small cohort of well-characterised NASH, that DNA methylation status of key fibrosis modifier genes loci in liver tissue can stratify patients according to fibrosis severity.^87^ Furthermore, a landmark study examined differential DNA methylation in 69 247 CpG sites in liver biopsies from mild (F0–2) versus patients with advanced (F3–4) fibrosis.^88^ The majority of differentially methylated sites became hypomethylated with disease progression (76%), whereas 24% underwent hypermethylation. Methylation correlated with 7% gene transcript levels such as FGFR2, a functioning receptor for keratinocyte growth factor, a protein made in chronic liver disease by stellate cells.^88^ A similar study was carried out in liver biopsies from lean controls, obese and patients with NASH. However, an important aspect of this study was the use of paired liver biopsies prebariatric and postbariatric surgery. This study showed that NAFLD-associated methylation changes to be partially reversible, and >400-fold enrichment in NRF1, HSF1 and ESRRA transcription factor binding sites.^89^ An important caveat to these studies is the analysis of DNA methylation in whole liver biopsies; the observed differences in DNA methylation density may simply reflect cellular or architectural changes inherent in the fibrogenic process. We have demonstrated that when areas enriched for myofibroblasts are separated from hepatocytes by laser capture microdissection, DNA methylation status at specific gene loci are distinct and specific,^90^ hence the analysis of DNA methylation in whole tissue has limited power for revealing mechanistic insights. A further caveat is that causality cannot be attributed to DNA methylation; longitudinal studies are needed to clarify if differences in DNA methylation at specific loci are predictive of progression to fibrosis. Concrete evidence of causality will require complex experimental investigations where specific methyl-CpG sites are manipulated in the context of a model of NASH-induced fibrogenesis .However, despite these caveats we can anticipate that the identification of sites of differential DNA methylation associated with fibrosis will lead to the development of new diagnostic biomarkers. Indeed, our laboratory have recently reported that fibrosis is associated with changes in DNA methylation density at the PPARγ promoter in circulating cell-free DNA isolated from patient plasma.^90^ Moreover, quantification of this epigenetic mark by pyrosequencing provided a powerful stratification tool, being able to clearly identify patients with NASH that had progressed to severe fibrosis.^90^ Further validation trials are ongoing with the aim of establishing the concept of quantification of DNA methylation from cell-free DNA as a liquid biomarker.

An emerging concept is the interplay between genetic and epigenetic variants in determining gene expression and NAFLD disease progression. As an example, an interesting study attempted to analyse both aberrant DNA methylation and its role in known genetic single nucleotide polymorphisms (SNPs) associated with severe NAFLD. The rs738409 Il148Met SNP in PNPLA3 is a well-known genetic modifier of NAFLD,^91^ although its regulation and function is currently unclear. This study found a differentially methylated region in the PNPLA3 promoter that was significantly hypermethylated in the livers of more severe (F3–4) fibrotic NASH, and was inversely correlated with mRNA levels in the same liver biopsy specimens, significantly with the GG genotype.^92^

A number of experimental studies have demonstrated control of hepatic lipid and carbohydrate metabolism by histone modifying enzymes. Normal circadian changes in lipid synthesis are associated with dynamic histone acetylation patterns of target genes in liver chromatin. This is controlled by histone deacetylase 3 (HDAC3) and its depletion promotes development of hepatic steatosis.^93^ HDAC3 can also integrate hepatic lipid and carbohydrate metabolism; mice with liver-specific depletion of Hdac3 had higher insulin sensitivity compared with WT mice, despite severe hepatic steatosis.^94^ This suggests a role for Hdac3 in reprogramming metabolites from usage in hepatic glucose production, to lipid synthesis and storage.^94^ Finally, the transcriptional activity of carbohydrate-responsive element-binding protein (ChREBP), a key transcriptional regulator of both lipogenic and glycolytic gene expression, is controlled by p300 which acetylates ChREBP on Lys672, enhancing its recruitment to its target gene promoters.^95^ Liver-specific p300 overexpression resulted in hepatic steatosis, confirmed in animal models of T2DM and obesity.^95^

The functions of ncRNAs in NASH have so far extended only to miRNAs, of which, many have been reported to be differentially expressed. miR-122 is well described regulator of lipid and cholesterol metabolism, accounting for 70% of the total liver miRNA pool, and is downregulated in NASH livers.^96^ More recently, there has been interest in whether miRNAs can serve as novel biomarkers of NAFLD. Elevated serum levels of miR-122, miR-34a and miR-16 were found in patients with NAFLD as compared with controls.^97^ miR-122 and miR-34a were both positively correlated with disease severity from steatosis to steatohepatitis.^97^ miR-122 was also correlated with liver enzymes, serum lipids and histological assessment.^98^ A recent study analysed a greater number of circulating miRNAs using a PCR-based array.^98^ Among 84 miRNAs analysed, circulating miR-122 was upregulated in NASH versus simple steatosis (3.1-fold) and controls (7.2-fold). Liver miR-122 was downregulated in NASH as prior studies demonstrated. Additionally, miR-192 and miR-375 were elevated in NASH serum compared with steatosis, and downregulated in NASH liver. A circulating miRNA signature of NAFLD appears attractive, but the predictive power of such a signature was only fair (AUROC ∼0.7).^98^

## Can liver disease be inherited?

Major advances in the field of transgenerational epigenetic inheritance have reopened a debate on the validity of Lamarck's original theory that species may adapt phenotype in response to environmental influences. It is only recently that studies in mammals have provided evidence that exposure to environmental stressors can drive stably inherited phenotypic adaptations in offspring that are inherited by epigenetic, rather than genetic mechanisms. Intriguingly, some of these studies concern the development of liver disease. For example, male inbred mice fed a low protein diet had offspring that increased liver expression of genes involved in lipid and cholesterol metabolism.^99^ Ancestral history of liver fibrosis in male outbred rats elicits a remodelling of DNA methylation in key fibrosis genes promoters, suppressing liver fibrosis in offspring.^100^ Importantly, comparable remodelling was demonstrated at similar loci in human fibrotic NAFLD liver tissue.^100^ Human studies in this field are lacking. However, a recent study in lean versus obese males found that spermatozoa had distinct sncRNA and DNA methylation signatures, particularly at loci controlling brain development and function, and that the epigenome could be remodelled after bariatric surgery, suggesting a mechanism for inheritance of metabolic dysfunction in progeny.^101^ There is also recent evidence for a heritable component to human NAFLD, ranging from 20% to 100%;^102^ ^103^ the contribution of epigenetic inheritance has recently been examined. A study conducted in monozygotic and dizygotic twins examined the relevance of miRNAs in discordant NAFLD phenotypes. In this study, 40 twin pairs underwent liver proton density fat fraction MRI to estimate liver fat content and serum miR profiling. Six twin pairs were discordant for NAFLD and a panel of 10 miRNAs discriminated between discordant pairs. Of these, miR331-3p and miR-30c were both highly heritable and targets included lipid and energy metabolism pathways.^104^ Future investigations aimed at clarifying the role of epigenetic imprinting in human liver disease are eagerly anticipated.

## Emerging epigenetic therapies

The discovery of novel, modifiable epigenetic targets has paved the way for the emergence of molecular-based epigenetic therapy; a table of registered trials of epigenetic drugs in liver disease is shown in table 1. The dynamism of the epigenome is relevant to drug development, as specific epigenetic alterations may be modified with treatment.

**Table 1:** Emerging epigenetic therapies in HCC. Clinicaltrials.gov accessed on 15th July 2016

Agent	Phase	Target	Primary outcome	Clinical trials identifier
SGI-110	II	DNMT	DCR at 16 weeks	NCT01752933
CUDC-101	Ib	HDAC/EGFR/Her2	AE	NCT01171924
Vorinostat	I	HDAC	MTD	NCT01075113
MRX34	I	miR-RX34	MTD	NCT01829971
PXD-101	I/II	HDAC	MTD/TR	NCT00321594
Resminostat	II	HDAC	PFSR at 12 weeks	NCT00943449

AE, adverse events; DCR, Disease Control Rate; DNMT, DNA methyltransferase; EGFR, epidermal growth factor receptor; HDAC, histone deacetylase; HER2, human epidermal growth factor receptor 2; MTD, maximum tolerated dose; PRSF, progression-free survival rate; TR, tumour response.

### miRNAs

Perhaps the best example of recent translational epigenetic therapy in liver disease is the miR-122 antagonist miravirsen, a locked nucleic acid-modified DNA phosphorothioate antisense oligonucleotide that sequesters mature miR-122 in a stable heteroduplex, inhibiting its function. Preclinical studies showed that miR-122 is essential for stability and propagation of HCV RNA.^105^ The 5′-UTR of HCV is highly conserved across all HCV genotypes^106^ and contains two closely spaced target sites, enhancing viral protein expression and protecting HCV RNA from nucleolytic degradation.^105^ In vivo studies in chimpanzees showed a marked suppression of plasma and liver HCV RNA,^107^ without evidence of resistant mutations at the two miR-122 binding sites in animal that received the highest dose of miravirsen. No adverse events were seen in phase I trials in healthy volunteers,^108^ leading to a phase IIa study in 36, treatment naïve, patients with non-cirrhotic chronically infected with HCV genotype 1. Dose-dependent reductions in HCV RNA levels were observed after 5 weekly administrations of miraversen, with no dose limiting adverse events.^24^ Notable benefits of miravirsen include a host factor antagonist with a high barrier to resistance, and likely cross genotype effect. Conclusions should however be tempered; germline deletion of miR-122 in mice resulted in steatohepatitis, and hepatocarcinogenesis.^109^ Also as observed in the phase IIa study, miR-122 antagonism lowered serum cholesterol levels demonstrating other miR-122 targets are affected during therapy.^24^ Although targeting miRNAs has shown promise in HCC,^110^ until more is known regarding the biology of miRNAs, a robust long-term therapeutic is not currently feasible for human HCC and liver fibrosis, and research is ongoing.

### HDAC inhibitors

The acetylation of histones is carried out by opposing enzymes, histone acetyl transferases and HDAC. HDACs catalyse the removal of acetyl groups from histone, compacting chromatin and silencing gene expression. Dysregulated expression of HDACs has been implicated in several cancers, including HCC. Overexpression of HDACs have been shown in 30%–50% of HBV-related HCCs,^111^ and overexpression of HDAC1 correlated with a high incidence of portal vein invasion, poor histological differentiation, more advanced TNM (tumour node metastases) stage and was an independent prognostic of HCC in patients after hepatic resection.^112^ Overexpression of HDAC3 is also correlated with advanced tumour stage and early recurrence after surgery.^113^ HDAC inhibitors (HDACi) have had favourable preclinical results, and induce apoptosis in tumour cells^114^ Pabinostat, a hydroxamic acid, pan-HDACi, when combined with sorafenib, demonstrated efficacy in HCC xenograft models.^115^ In humans, efficacy of HDACi has been demonstrated in advanced cutaneous and peripheral T-cell lymphoma.^116^ A recent phase I/II clinical trial of resminostat in 57 patients with HCC and radiological progression on sorafenib treatment was recently reported; median time to progression and overall survival were 6.5 and 8.0 months for combination therapy.^117^ The results from resminostat monotherapy were dismal, but suggest that it may restore sensitivity to sorafenib, and be a potential therapeutic option.^117^ No safety issues were reported in this study, although the most common adverse events were GI upset, thrombocytopenia and fatigue. Another prior phase I/II clinical trial of belinostat monotherapy for advanced HCC reported disappointing progression-free survival 2.6 months and overall survival of 6.6 months.^118^ In vitro and in vivo studies suggest that HDAC are also upregulated in chronic fibrotic liver disease.^119^ HDACi block myofibroblast transdifferentiation and fibrogenesis in animal models.^119^ ^120^ Currently, no clinical trials have evaluated the efficacy of HDACi in human chronic liver disease; deeper understanding of HDAC substrate, activities and the molecular mechanisms underpinning HDACi inhibition is necessary for development of inhibitors with greater specificity. Notably, histones are the substrate for HDACs and act as acetyl-lysine erasers on non-histone proteins such as p53, NF-κB subunits and cytoskeleton proteins.^121^ There are several 2nd generation HDACi in development, targeting individual HDACs rather than promiscuously; it is hoped that they may shed light on the mechanisms underlying individual HDACs, and also mitigate the toxicity associated with current HDACi.^122^

### HDAC activators

SIRT1 is a class III HDAC; its action is nicotinamide adenine dinucleotide (NAD+)-dependent, and is regulator of metabolism and ageing,^123^ hallmarks of NAFLD. As mice age, liver expression of SIRT1 is reduced.^124^ When mice with liver-specific knockout of SIRT1 are challenged with a high fat diet, hepatic steatosis, inflammation and endoplasmic reticulum stress results due to altered PPARα signalling.^125^ Equally, mice treated with SIRT1 activators such as resveratrol were prevented from developing diet-induced NAFLD.^126^ ^127^ In obese humans, liver expression of SIRT1 increased after extensive weight loss.^128^ However, patients with NAFLD treated with 8 weeks of resveratrol did not improve hepatic steatosis (as measured by MRI) or insulin resistance, when compared with placebo.^129^ Indeed, markers of hepatic inflammation such as alanine aminotransferases increased.^129^ Thus, further studies are needed to clarify a role for SIRT1 activators in patients with NAFLD.

### DNMT inhibitors

Targeting aberrant DNA methylation in HCC using DNMT inhibitors is currently being tested in phase II trials. Guadecitabine (SGI-110) is a stabilising dinucleotide comprising deoxyguanosine and the DNA demethylation agent decitabine (2-deoxy-5′-aza-cytidine), currently an Food and Drug Administration-approved therapy for myelodysplastic syndrome. Experimental data suggest that combination therapy with SGI-110 may sensitise HCC cells to oxaloplatin.^130^ In xenograft studies performed in athymic nude mice, combination therapy reduced tumour growth and decrease on Ki67 levels, suggesting reduced cell proliferation.^130^ Results from a now complete phase II clinical trial are now awaited. Many experimental studies have identified DNA methylation as a potential target in the treatment of chronic fibrotic liver disease. However, there are currently no clinical trials testing DNMTi in liver fibrosis.

## Conclusions

**Figure 2:**
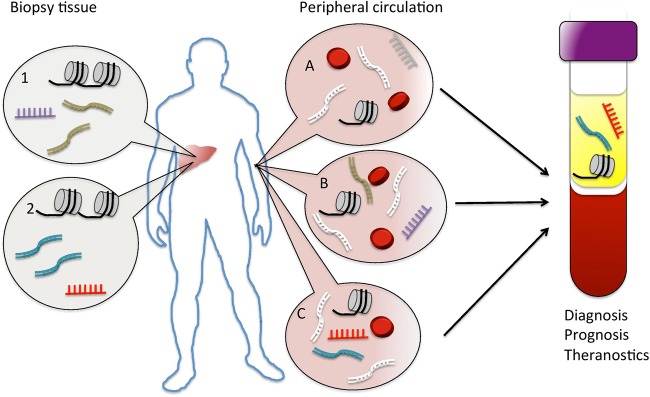
Potential clinical applications of epigenetic mechanisms. Dysregulated epigenetic mechanisms are characteristic of both (1) liver cancer and (2) liver fibrosis and are readily examined from biopsy tissue. (A) Cell-free DNA, extracellular microRNAs and nucleosomes from necrotic/apoptotic cells can normally be detected in the circulation, of which the liver provides a proportion; aberrant epigenetic signatures associated with (B) cancer and (C) fibrosis are released into the circulation, potentially providing blood-based markers that could be used to aid diagnosis, prognosis and theranostics.

This review aims to highlight key areas in which epigenetic mechanisms influence liver disease phenotype by effecting the underlying cellular biology, and potential clinical utility, as summarised in figure 2. Advances in non-invasive risk stratification are beginning to be realised, and new epigenetic drugs are being developed and tested in clinical trials. The current challenge for hepatologists in providing effective healthcare for their patients is the need for better diagnostics, prognostics and therapeutics, personalised to those who would most benefit. While genetic factors are undoubtedly important in this endeavour, the opportunity provided by better understanding and exploitation of the fine-tuning, epigenetic mechanisms operating in liver disease, promises to drive forward an unprecedented advance in precision medicine.
